# Experimental designs for small randomised clinical trials: an algorithm for choice

**DOI:** 10.1186/1750-1172-8-48

**Published:** 2013-03-25

**Authors:** Catherine Cornu, Behrouz Kassai, Roland Fisch, Catherine Chiron, Corinne Alberti, Renzo Guerrini, Anna Rosati, Gerard Pons, Harm Tiddens, Sylvie Chabaud, Daan Caudri, Clément Ballot, Polina Kurbatova, Anne-Charlotte Castellan, Agathe Bajard, Patrice Nony

**Affiliations:** 1 Hôpital Louis Pradel, Centre d’Investigation Clinique, INSERM CIC201/UMR5558, 28, Avenue du Doyen Lépine, Bron 69677 cedex, France; 2 CHU Lyon, Service de Pharmacologie Clinique, 8 rue Guillaume Paradin, Lyon, BP8071, 69376 cedex 08, France; 3 University of Lyon 1, UMR 5558, CNRS Lyon, 8 rue Guillaume Paradin, Lyon, BP8071, 69376 cedex 08, France; 4 Senior Expert Statistical Methodologist, Novartis Pharma AG, Basel CH-4056, Switzerland; 5 INSERM U663 «Epilepsy in childhood and brain plasticity », Hopital Necker - Enfants Malades, Paris, France; 6 Univ Paris Diderot, Sorbonne Paris Cité, 75019, Paris, France; 7 AP-HP, Hôpital Robert Debré, Unité d'Epidémiologie Clinique, 75019, Paris, France; 8 Inserm, CIE5, 75019, Paris, France; 9 Pediatric Neurology Unit and Laboratories, Children’s Hospital A. Meyer-University of Florence, Florence, Italy; 10 University Paris Descartes, Inserm UMR 663, Paediatric Committee -EMA (London UK), Head PIP WP (AFSSAPS), Cochin - Saint Vincent de Paul Hospital, Paris, France; 11 Erasmus University Medical Center, Sophia Children’s Hospital, P.O. box 2060, Rotterdam 3000 CB, The Netherlands; 12 Unité de Biostatistique et d’Evaluation des Thérapeutiques, Centre Léon Bérard, Lyon 69373, France

## Abstract

**Background:**

Small clinical trials are necessary when there are difficulties in recruiting enough patients for conventional frequentist statistical analyses to provide an appropriate answer. These trials are often necessary for the study of rare diseases as well as specific study populations e.g. children. It has been estimated that there are between 6,000 and 8,000 rare diseases that cover a broad range of diseases and patients. In the European Union these diseases affect up to 30 million people, with about 50% of those affected being children. Therapies for treating these rare diseases need their efficacy and safety evaluated but due to the small number of potential trial participants, a standard randomised controlled trial is often not feasible. There are a number of alternative trial designs to the usual parallel group design, each of which offers specific advantages, but they also have specific limitations. Thus the choice of the most appropriate design is not simple.

**Methods:**

PubMed was searched to identify publications about the characteristics of different trial designs that can be used in randomised, comparative small clinical trials. In addition, the contents tables from 11 journals were hand-searched. An algorithm was developed using decision nodes based on the characteristics of the identified trial designs.

**Results:**

We identified 75 publications that reported the characteristics of 12 randomised, comparative trial designs that can be used in for the evaluation of therapies in orphan diseases. The main characteristics and the advantages and limitations of these designs were summarised and used to develop an algorithm that may be used to help select an appropriate design for a given clinical situation. We used examples from publications of given disease-treatment-outcome situations, in which the investigators had used a particular trial design, to illustrate the use of the algorithm for the identification of possible alternative designs.

**Conclusions:**

The algorithm that we propose could be a useful tool for the choice of an appropriate trial design in the development of orphan drugs for a given disease-treatment-outcome situation.

## Background

Small clinical trials are necessary when there are difficulties in recruiting enough patients for conventional frequentist statistical analyses to provide an appropriate answer. These trials are often necessary for the study of rare diseases as well as specific study populations e.g. paediatric, geriatric, individually tailored therapies, regional subpopulations. In these settings the issue of small sample size has to be faced. The European Medicines Agency guidelines on clinical trials in small populations (CHMP/EWP/83561/2005) considers the problems associated with clinical trials when there are limited number of patients available to study and clearly defines the field of application [1].

Rare diseases are defined on the basis of their low prevalence, i.e. less than 1 in 2,000 people affected. It has been estimated that there are between 6,000 and 8,000 rare diseases that may affect up to 30 million people in the European Union alone, although these figures do not come from published peer reviewed epidemiological studies [2,3]. Only about 250 of these diseases have a code in the existing International Classification of Diseases (ICD) (10th version) [4]. Rare diseases cover a broad diversity of diseases and patients, with about 50% of those affected being children. About 80% of these rare diseases have an identified genetic origin involving one or several genes or chromosomal abnormalities [5]. The others are caused by infections (bacterial or viral), or allergies, or are due to degenerative, proliferative or teratogenic (chemicals, radiations, etc.) causes. Some rare diseases are also caused by a combination of genetic and environmental factors [5]. Drugs (including orphan drugs) are developed for treating these rare diseases, and their efficacy and safety need to be evaluated but due to the small number of potential trial participants, a standard randomised controlled trial is often not feasible [6].

In children the issue is not restricted solely to rare diseases as the difficulty in recruiting sufficient numbers of patients is a problem for even frequent diseases. This difficulty is mainly due to ethical and psychological considerations, which not only represent an obstacle to running clinical trials but also to protecting the children. These considerations need to be taken into account to design trials which minimise the risk for individual patients (e.g. minimal numbers of samples in pharmacokinetic/pharmacodynamic studies) as well for the whole paediatric population [7]. Consequently, the use of innovative methodologies enabling fewer patients to be recruited could become the rule for dose-finding and efficacy studies in the future.

Clinical trial methodology has evolved since the mid-20^th^ century so that now well-established and validated methods are available for the design, conduct and analysis of clinical trials [8]. It is generally accepted that an appropriate trial design includes a sufficiently large sample size and statistical power, and methods for minimising bias to enable the results to be reliably interpreted. The randomised, parallel-group controlled clinical trial design is generally considered as the gold standard, but in some situations it is difficult to use this design. The type of situation when it is not feasible includes rare diseases with very low incidence/prevalence, individually tailored therapies, and specific trial populations. The general requirements for small trials are the same as those for adequately sized trials, i.e. their design and analysis should enable a reasonable measure of the treatment effect to be obtained. The design should include an outcome that can be measured to determine change or ‘success’, via a baseline value and an ‘under-treatment’ value for the outcome.

The minimisation of systematic bias remains fundamental, as for the more classical trial designs. These biases include: selection bias, which is the biased allocation of patients to treatment or placebo groups; performance bias, which is the unequal provision of care apart from the treatment under evaluation; detection bias, which is the biased assessment of the outcome; attrition bias, which is the biased occurrence and handling of deviations from protocol and loss-to-follow-up. These biases can be minimised using validated methodology. Good-quality central randomisation can minimise selection bias. Double-blind follow-up and outcome evaluation can minimise the other biases, and when this is not possible, the trial outcome should be measured in a blinded manner, by someone who is not involved in the patient’s care. Specific methods for the management of missing data exist, e.g. replacement of missing measurements in designs with intra-individual assessments, and intention-to-treat analyses. A specific statistical analysis plan is necessary for all trial designs, and should be defined, *a priori*, in the trial protocol; the analysis plan should be coherent with hypothesis tested and should include appropriate control of the type I error rate [8].

There are a number of trial designs that have been proposed as alternatives when the usual parallel group designs are not appropriate or feasible [9]. Each of these designs offers specific advantages, but they also have specific limitations. Thus the choice of the most appropriate design is not simple. In addition, for any given situation, several designs may be possible. We performed a literature review of alternative trial designs and we summarise their main characteristics in this paper and present an algorithm that can be used to select the most appropriate design(s) for given disease-drug-outcome situations. To illustrate the use of the algorithm, we will discuss case studies of published clinical trials, to ascertain if alternative study designs could have been used.

## Methods

PubMed was searched using combinations of the terms given in Table 1 in the title field, with no limitations in terms of language published up to end of 2010, to identify publications about the characteristics of different trial design methods that can be used in randomised, comparative small clinical trials, other than the standard randomised controlled trial design. In addition, the tables of contents for 11 journals were hand-searched; the years for each journal are indicated in Table 1.

**Table 1:** Search strategy for the identification of articles on the methods used for small clinical trials


**Terms combined in PubMed search**	
**•**	(“Rare diseases” OR “orphan*”) AND (“Epidemiologic Methods” OR “Research Design” OR “Clinical Trials as Topic”)
**•**	Rare disease*
**•**	Clinical trial*
**•**	**Clinical research**
**•**	**Withdrawal**
**•**	Winner*
**•**	Loser*
**•**	**Sequential**
**•**	**Adaptive**
**•**	**Delayed start**
**•**	**Early escape**
**•**	**N-of-1**
**•**	Randomi*
**•**	**Placebo phase**
**•**	**Three stage**
**Journals (years) hand-searched:**	
**•**	**Statistics in Medicine (1990–2010)**
**•**	**Orphanet Journal of Rare Diseases (2006–2010)**
**•**	**Controlled Clinical Trials (2000–2004)**
**•**	**Contemporary Clinical Trials (2005–2010)**
**•**	**BMC Medical Research Methodology (2001–2011)**
**•**	**Journal of Biopharmaceutical Statistics (1991–2012)**
**•**	**Biometrical Journal (1990 – 2012)**
**•**	**Statistica Sinica (1991–2012)**
**•**	**Journal of Statistical Planning and Inference (1990 – 2012)**
**•**	**Journal of the American Statistical Association (1990–2012)**
**•**	**American Journal of Biostatistics (2010 – 2012)**

* = truncation symbol used in PubMed search.

The characteristics of the identified trial designs and their advantages and disadvantages were summarised. The assessment of advantages and limitations of each design was based on the experience of the authors and that of experts and academic opinions. Based on these characteristics, we identified decision nodes, and then developed an algorithm that can be used in practice to select the most appropriate trial design.

## Results

### Results from literature search

A total of 1420 abstracts were identified. After screening the titles and abstracts and obtaining full papers for selected articles we identified a total of 75 publications that reported information about the methods for various randomised, comparative trial designs that could be used in for the evaluation of therapies in orphan diseases.

### Summary and general characteristics of randomised, comparative trial designs used in practice

**Figure 1:**
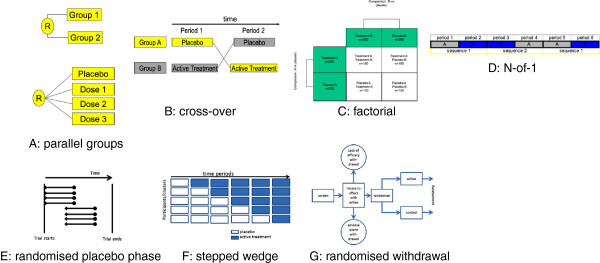
Schematic representation of some randomised clinical trial designs.

The main characteristics and the advantages and limitations of the 12 trial designs (adaptive randomization designs were grouped in one single category) that were identified are summarised in Figure 1 and Table 2[10-24]. Some examples of trials using the different designs are given in Table 3[12,25-49].

**Table 2:** Summary of the characteristics of the various randomised, comparative trial designs (it is assumed for all designs that the control group is a placebo)

**Study design**	**Main characteristics**	**Randomisation**	**Main advantages**	**Main disadvantages**
**Parallel groups** **(comparison between groups)**[24]	Patients are assigned to a treatment group for the duration of the trial.	Randomisation to one of two or more treatment groups, with a pre-specified randomisation ratio.	Design simple to understand and to implement.	Larger sample size often required, compared with other designs.
			Treatment groups can have different numbers of patients.	
				Difficulties with recruitment possible, if placebo-controlled.
			Analysis and interpretation of results is simple.	
				Cannot estimate the contribution of inter- and intra-patient variability to the overall variability.
**Factorial**	Can answer two or more questions with one trial	Patients are randomised twice, once for treatment A or placebo and then for treatment B or placebo	Time-saving for the trial sponsor	Need to be sure that there is no interaction between treatments A and B
			Requires fewer patients to obtain the answer to two or more questions	
**Cross-over**	Patient receives both of two treatments, A and B, in a pre-specified sequence. Patients act as their own control.	Randomisation to a pre-specified treatment sequence.	Smaller sample size than parallel groups.	Stable chronic diseases (assumes patient’s state is comparable at the start of both periods of treatment). Endpoint must not be sensitive to learning processes. Requires a wash-out period between treatment periods. Follow-up is at least twice as long compared with corresponding parallel group trials. The analysis must confirm the absence of treatment - period interaction
			Results depending only on within-patient variability.	
			Often used in healthy volunteers (for phase 1 clinical trials)	
**Latin square**	More than two treatments to compare	Randomisation to a pre-specified treatment sequence.	Same as for cross-over design.	Same as for cross-over design, except carry-over is controlled (similar properties as those for Latin square design).
	Balanced design, i.e. every treatment (or dose) appears only once in each sequence and each treatment period.			
**N-of-1**[14,19,23]	Only one patient and design aims to assess effects of several treatments in one individual	The order of treatment(s) and placebo periods are randomly assigned for the patient	Provides an estimate of individual effectiveness (personalized medicine)	Same as for cross-over design. Needs a stable, chronic disease
			Patients are more likely to have better adherence to treatment, and understand their disease and treatment better	
**Delayed start**[10,12,16]	Two phases: initial placebo controlled phase (patients randomised to treatment or placebo) followed by active control phase (all patients receive treatment) – those in the initial placebo group have a delayed start	In first phase, patients randomised to early start group (treatment) or delayed start group (placebo)	Allows more patients to receive active treatment	At the start of the second phase, the patients are not comparable. No real blinding for the second period; carry over effect possible.
			Can distinguish effects on symptoms and effects on the disease evolution	
**Randomized placebo-phase**[13]	All patients receive the tested treatment in the end – but have varying lengths of time on placebo.	Randomisation of time from enrolment to starting tested treatment	Can be used for disease-modifying therapies, in diseases with a rapid, unfavourable evolution. All patients receive active treatment	Variable length of placebo period reduces statistical power
	Assumes that a response will occur sometime after an effective treatment is given, so that patients who start the treatment earlier should, on average, respond sooner			
				Low and intermediate potency therapies show large variability for response
				Limited ability to estimate size of treatment effect
**Stepped wedge**[11]	All patients receive tested treatment in the end. Intervention allocated sequentially to participants (either as individuals or clusters of individuals)	For a 5-step wedge design, all patients start with control then for the following five time periods individual or clusters randomised to treatment to finish in the last period with all patients receiving tested treatment	Useful when there is a prior belief that treatment will do more good than harm	There might be a risk of contamination between intervention participants, and a need for blind assessment of outcome
			Also, when an innovation cannot be delivered concurrently to all units	
**Randomised withdrawal**	Used to assess treatment continuation in patients who are responding to the treatment.	Randomisation of responders to continue treatment or switch to placebo	Reduces the time on placebo since only responders are randomised to placebo.	For use in chronic diseases, Not suitable for unpredictable diseases (e.g. spontaneous remission) or those with slow evolution. The treatment effect is overestimated since only responders are included (and compared to placebo)
	All patients initially receive the tested treatment; responders are randomised to continue treatment or to receive placebo			
			Can assess if treatment needs to be continued or can be stopped	
				Possible carry-over effect for adverse effects.
**Early escape**	Patients withdrawn if they satisfy *a priori* failure criterion	Randomisation to active treatment or placebo	Reduces the time on placebo or in treatment failure.	Difficult to define a binary failure/success outcome.
			Analyse failure rate, so minimises exposure to ineffective treatment	
				Only short-term efficacy evaluated.
				Loss of power if significant number of patients ‘escape’
**Three-stage**[15]	Initial randomised placebo-control phase, a randomised withdrawal stage for responders, and a third randomised phase for placebo non-responders who subsequently respond to treatment	Randomisation to treatment or placebo and randomised withdrawal for responders	Three separate (independent) assessments of efficacy which are then combined (Fisher’s method) to derive a single overall p-value.	Applicable only to chronic conditions where both response to therapy and withdrawal of therapy can be assessed.
				Care should be taken to allow the withdrawal phase to be sufficiently long so that the drug can be completely washed out and the clinical effects of therapy reversed.
				Subjects may barely meet criteria for being a responder and would consequently forgo active treatment even though they may have benefited from it.
				Since fewer patients may be available in the initial stage of the trial, the ability to precisely determine initial response rates may be less than with a traditional randomized trial design.
				May be less suited for controlled assessment of safety
			Fewer patients required compared with parallel group design.	
			Reduces the time on placebo or non-efficacious treatment.	
			May evaluate the efficacy of a therapeutic agent in a particular patient subpopulation when efficacy in the general patient population has already been established.	
**Adaptive randomization designs -play the winner**[17,20]	An adaptive randomization design.	The probability of being randomised to one group is modified according to the results obtained with previous patients. It favours the group with favourable results (play the winner), or penalise the group with unfavourable results (drop the looser); it can be generalised to multi-treatment clinical trials, and delayed responses (Generalized drop the looser)	Reduces the number of patients receiving a less effective treatment.	Unequal sample size reduces power.
	Need to have binary outcome, (success/failure)			In some situations, the number of patients who have actually received one of the treatments is very low.
			Could improve patient recruitment due to better satisfaction	
**-drop the losers**[18]				
**-generalised drop the loser**[21,22]				

**Table 3:** Examples of clinical trials that have used the different designs

**Study design**	**Examples**
**Parallel groups**	• Phosphodiesterase-5 inhibition for pulmonary hypertension in heart failure [31]
	• Vigabatrin in infantile spasms due to tuberous sclerosis (comparative parallel design) [27]
	• Stiripentol in Dravet syndrome (placebo controlled parallel design) [28]
**Factorial**	• Aspirin and simvastatin for pulmonary arterial hypertension [35]
**Cross-over**	• Amantadine in Huntington disease [40]
	• Oral sildenafil therapy in severe pulmonary artery hypertension [45]
	• Sirolimus therapy to halt the progression of ADPKD [42]
**Latin square**	• Plasma exchange for induction and cyclosporine A for maintaining remission in Wegener’s granulomatosis [47]
	• Assessment of disease flare in patients with systematic lupus erythematosus [33]
**N-of-1**	• Amitriptyline in fibromyalgia [34]
	• Tramadol to treat chronic cough [37]
	• L-arginine in ornithine transcarbamylase deficiency carrier [32]
**Delayed start**	• Rasagiline in Parkinson’s Disease [16,41]
**Randomised placebo-phase**	• Low dose phenelzine in the chronic fatigue syndrome [39]
**Stepped wedge**	• Long-term efficacy of HBV vaccine to prevent liver cancer and chronic liver disease [49]
	• School-based anti-smoking campaign, (delivered by one team of facilitators who travel to each school)
	• Sure Start programme in the UK (http://www.ness.bbk.ac.uk)
**Randomised withdrawal**	• Withdrawal of hydroxychloroquine sulfate in systemic lupus erythematosus [48]
	• Etanercept in children with polyarticular juvenile rheumatoid arthritis [38]
	• Vigabatrin withdrawal randomized study in children with epilepsy [26]
	• Antipsychotic withdrawal with Alzheimer’s Disease [25]
	• Stiripentol withdrawal design in children with partial epilepsy [29]
**Early escape**	• Intravenously golimumab in patients with active rheumatoid arthritis [36]
	• Pain control for post-operative pain [46]
**Three-stage**	• Etanercept in children with polyarticular juvenile rheumatoid arthritis [15,38]
**Adaptive randomisation**	• Prevention of postoperative venous thromboembolism in digestive surgery [43,44]
	• Reduction of maternal-infant transmission of Human Immunodeficiency Virus Type 1 with zidovudine [30]
	• No example found for the ‘drop the loser’ design

### Parallel group design

In a parallel group design trial, individuals are randomized to receive the tested treatment or control. This is the most commonly used design, which is possible in almost any situation, but requires larger sample sizes than other designs [24].

### Factorial design

With the 2 × 2 factorial design trial, participants are randomized to treatment A or corresponding placebo to test one hypothesis, and randomized again within each group to treatment B or corresponding placebo to test a second hypothesis, thus enabling two different hypotheses to be tested simultaneously. This design is based on the parallel group design. It also requires that there is no interaction between treatments A and B. If interaction exists, then loss of power is possible in case of separate analyses of the four different combinations. This design enables the measurement of an effect or an interaction which otherwise might not be apparent.

### Cross-over design, Latin square, N-of-1

Each participant in a cross-over trial receives two treatments in a random order and acts as their own control. Latin-square design differs from cross-over design in terms of the number of studied treatments; latin-square design is used when more than two treatments are compared in the same trial. For example when three treatments are considered in the trial, the corresponding latin-square involves three treatment periods and two wash-out periods occurring between each treatment period for each of the three groups of patients.

N of 1 trials or single-subject designs are defined as time-series designs in which an intervention is evaluated in one single patient. A typical single patient trial consists of experimental/control treatment periods repeated a number of times. The order of treatment is randomly assigned within each treatment period pair. Formally, this design is known as a structured within-patient randomized controlled multi-crossover trial design. Usually, the primary objective of such a trial is to determine the treatment preference for the individual patient.

For cross-over trials, as for all intra-patient designs, the disease must be stable, and the patient’s health status must be identical at the beginning of each treatment period. There can be a carry-over effect, if the treatment effect from the previous period is still present during the following period. To avoid this, a wash out period is generally added between each treatment period of the trial. The duration of follow-up for the patient is therefore longer than for a parallel design, and there is a risk that a significant number of patients do not complete the study.

### The delayed start design

In this design an initial randomised placebo controlled phase is followed by a phase during which all patients receive the active treatment. This design can be used to assess disease progression as well as disease relapses (or other short term outcomes). This trial design requires that the treatment periods are sufficiently long for a therapeutic effect to be obtained, that as few as possible patients are lost-to-follow-up (and if possible, the same number in both groups) and that there are a sufficient number of follow-up visits to measure the treatment effect to allow a precise estimation of the treatment effect slope. The limitations of this design include the fact double-blinding is only really present in the first trial period, since with this design in the second period all the patients receive active treatment. In addition, the evolution of the symptoms during the follow-up can enable the treatment group for the previous period to be identified. This can induce an evaluation bias. A carry-over effect from the first to the second period cannot be excluded, as well as a training effect if the primary criterion is a score. Hence, this type of trial is almost always explicative (i.e. evaluates the effect of the treatment on the symptoms and the evolution of the disease), losing all its pragmatic repercussions, unlike, for example, a classical parallel group trial with a follow-up equivalent to the two periods in the delayed start design.

### Minimising time on inactive treatment or placebo: randomised withdrawal, early escape, randomised placebo phase, stepped wedge designs

With the randomised withdrawal design, all eligible patients with the disease being studied receive open-label treatment for a specified period to identify a subgroup of patients who can successfully achieve a pre-defined level of response. The patients in this subgroup are then randomized to continue the tested treatment or to receive a placebo in a double-blind fashion. The randomised withdrawal design aims to evaluate the optimal duration of a treatment in patients who respond to the treatment. In the randomised early escape design, for the patients who do not respond to therapy, time on ineffective treatment is minimised. Both these designs are combined in the three-stage randomised trial design. In the other possible designs (randomised placebo phase, stepped wedge trials) the time spent on placebo is minimised, and all patients receive the active treatment at the end.

### Adaptive randomisation (play the winner, drop the looser designs)

The play-the-winner and the drop-the-loser designs aim to favour the group with the best chance of success by increasing the probability of patients being randomised to that group. For adaptive randomization designs, the procedure is best described by using the urn model which is common in the statistical literature; in the urn there are various types of balls representing particular treatments; patients accrue sequentially and at each stage, the probability of allocating a particular treatment to a given patient depends on the number of various types of balls in the urn. The response of each patient after treatment plays an essential role in the determination of subsequent compositions of balls in the urn.

In the randomized play the winner (PW) procedure, the basic strategy is to ‘reward’ more balls to successful treatments. The urn contains K different types of balls, representing K different treatments. When a patient arrives, a ball is drawn at random with replacement. If it is a type *i* ball, the patient receives treatment *i*. A successful response to the treatment results in the addition of a type *i* ball to the urn. If the response is a failure, a different ball is added to the urn, this ball being partitioned according to the existing proportion of balls for other treatments in the urn.

In the drop the loser (DL) procedure, instead of adding balls to reward successes, balls are removed when failures are observed. In the urn, besides treatment balls, there are immigration balls. When an immigration ball is selected, balls for all types will be added (except immigration), preventing the total elimination of any type of treatment balls. The DL rule was reported to have small variability in terms of treatment attribution and high statistical power and has been shown to yield satisfactory results in terms of reducing the number of failures. Nevertheless, adaptive randomization has some limitations, i.e. a lack of clear methodology to cope with delayed test responses which are common in clinical studies and its application is limited to clinical trials with binary responses.

### Decision nodes

The decision nodes were empirically derived from the requirements and limitations of each specific design as well as from their advantages. We identified the design characteristics that seemed most likely to guide the choice of a specific design:

•reversible or irreversible outcomes

•fast (defined as up to a few weeks) or slow response to treatment

•possibility of minimising the time on placebo

•possibility that all patients received active treatment by the end of the trial

•possibility of performing intra-patient or inter-patient comparisons.

### Algorithm development and testing

**Figure 2:**
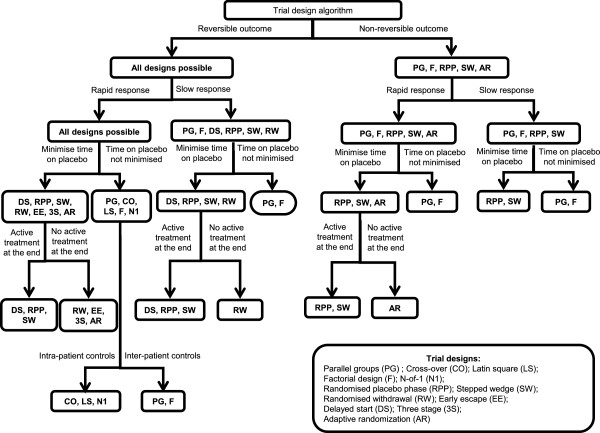
Schematic representation of trial design algorithm.

These decision nodes were used to design the algorithm (Figure 2). To test this algorithm, we took some examples of clinical trials that used one of these designs and worked through the decision nodes to see what alternative designs would have been possible.

#### Case study 1

The first example involves an n-of-1 trial that assessed the efficacy of L-arginine vs placebo in a patient with ornithine transcarbamylase deficiency (OTCD) [32]. Female carriers of this autosomal genetic disorder may be asymptomatic, or have symptoms ranging from protein aversion only, to profound neurological impairment and death due to secondary encephalopathy. Arginine supplementation is required, but it is not certain if mildly symptomatic females will benefit from this treatment. An n-of-1 trial in a mildly symptomatic woman with alternative weeks of placebo and L-arginine was performed. The patient’s symptoms were measured on days 5 and 6 of each weekly period; three treatment pairs of L-arginine and placebo, each with two measurements, were used to minimise bias. The results showed consistently higher scores for L-arginine than for placebo. The outcome in this example is reversible, and the response is rapid so, using the algorithm, all designs would be possible (left-hand side of the decision tree). At the third decision node, if it was decided to minimise time on placebo, seven different designs would remain possible. Since this treatment is intended as a long-term treatment, and in view of the encouraging results from this n-of-1 trial, larger scale trials could be designed to ensure that all patients received active treatment by the end of the trial; in this context, delayed start, randomised placebo phase and stepped wedge designs could be considered.

#### Case study 2

Another example that was used to test the algorithm was a randomized, blinded withdrawal trial of intravenous immunoglobulin in patients with polyarticular juvenile rheumatoid arthritis resistant to other treatments [50]. The outcome was ‘clinically important improvement’, which is reversible, but relatively slow (>3 months). If the investigators had wanted to minimise time on placebo, they could have used the delayed start, randomised placebo phase or stepped wedge designs. The randomized withdrawal design is suitable for a chronic disease. In this example, the authors justified the choice of trial design on the grounds of ethics; reducing the time on placebo (and preventing long-term harmful effects due to worsening of the disease).

#### Case study 3

A trial with a play-the-winner design assessed the efficacy of enoxaparin given before or after digestive surgery to prevent venous thromboembolism [51]. In this trial, the outcome, venous thromboembolism, is irreversible and the response under treatment is rapid. In addition, both groups received active treatments, since the time of treatment start, before or after surgery, was randomised. In the algorithm, we can see that four other trial designs could have been used. However, in this context, some of the designs would not be possible; e.g. randomised placebo phase, and stepped wedge. Using a parallel group or factorial trial design for simultaneous comparison of two treatments with each of their controls (provided there is no interaction) would have been possible.

#### Case study 4

The final example is a trial with a delayed start design to assess a potentially disease-modifying neuro-protective drug, rasagiline in patients with Parkinson’s disease [16]. The primary endpoint was based on the Unified Parkinson’s Disease Rating Scale (UPDRS; a 176-point scale with higher numbers indicating more severe disease); this outcome is reversible and the response can be considered to be slow. Possible designs are: randomised placebo phase, stepped wedge design, both of which would have also minimised time on placebo and ensured that all patients received active treatment in the end. However, the selected design is the only one able to measure the treatment effect on the symptoms and the evolution of the disease. Three hypotheses had to be tested (sequentially), in order to conclude that the treatment was efficacious in this trial:

1. the superiority of the treatment over placebo: first period, between weeks 12 and 36;

2. the superiority of the early start over the deferred start (comparison of the difference in effect over the combined periods 1 and 2, week 72 vs. baseline; and

3. the non-inferiority of early vs. deferred start (comparison of the effect slopes in the second period, between weeks 48 and 72.

## Discussion

In this review of alternative clinical trial designs for the evaluation of interventions in the setting of rare diseases we have identified 12 possible designs. Based on the characteristics of these trial designs we have developed an algorithm and have illustrated its use through examples of published trials. These examples show that alternative designs to those used in the publications would have been possible. Factors, such as objective(s) of the trial, number of patients needed, length of trial, and how the variability is handled, could be important in the choice of the most suitable trial design. A recently published review provided an algorithm with six alternative designs [52]. Although this seems to be a simpler approach to decision-making than our approach, our algorithm includes 12 alternative designs, all of them being randomised designs.

One limitation to our algorithm is that we have arbitrarily selected decision nodes to go through the algorithm but other nodes are possible, for example, stable disease or not. These proposed decision nodes were selected based on the experience of two of the authors (CC and PN) and are not based on objective criteria. This proposition can be debated by the scientific community and will need to be tested before it can be validated.

In this paper, we addressed the design of a small pivotal trial where one experimental treatment is compared with a control. We did not address the design of clinical programmes for rare diseases, seamless approaches which can combine dose selection and confirmation in the same trial, or dose (and regimen) finding trials [53-55].

Other approaches, that we can call ‘meta-methods’ or ‘orthogonal methods’ can minimise the number of patients needed if applied to some of the ‘basic’ designs considered in our algorithm. For example, meta-analyses of clinical trials, including prospective meta-analyses, Bayesian inferential methods, statistical techniques such as sequential analyses (e.g. triangular tests) and sample size reassessment methods could contribute to minimise the sample size. However, the fact that sample size reassessment could contribute to minimize the sample size is theoretical, as common practice is to use sample size reassessment to increase rather than decrease sample size (but when used with group sequential boundaries, the design as a whole can contribute to diminish the sample size).

Based on the algorithm that was developed we can see for any given disease-outcome situation that there is generally more than one design that could be used. Other factors could be incorporated into the selection of the most appropriate design, such as statistical power, trial duration for patients, investigators and trial sponsors, and more generally the costs involved. This second stage in the decision process will require building models of the pair: disease-treatment that be used to simulate the results from each design before selecting the best design for the specific research question. This approach will be developed in the setting of the CRESim project (Rare disease: use of clinical trial simulation for the choice and optimisation of study design), funded by the European Commission PRIOMEDCHILD ERA-NET Programme [56]. One deliverable from this project will be the development of a web-based platform for performing *in silico* experiments to assess different designs for drug evaluation in children with rare diseases. The algorithm could also be useful in other settings, such as specific small sub-populations of common diseases and in settings where recruitment is likely to be very difficult.

## Conclusions

The algorithm that we propose seems to be a useful tool in the case of rare diseases and the development of orphan drugs as well as for specific populations where recruitment could be difficult. Use of this algorithm will facilitate the choice of the most appropriate design for a given disease-treatment-outcome situation.

## Competing interests

The authors declare that they have no competing interests.

## Authors’ contributions

All the authors contributed to the conception of this project and the analysis and interpretation of the trial designs in the setting of the CRESim and Epi-CRESim project groups. They were all involved in critically revising the manuscript for important intellectual content and they have all approved this final version.
